# The comorbidity of increased arterial stiffness and microalbuminuria in a survey of middle-aged adults in China

**DOI:** 10.1186/s12872-018-0817-1

**Published:** 2018-05-04

**Authors:** Rujia Miao, Liuxin Wu, Ping Ni, Yue Zeng, Zhiheng Chen

**Affiliations:** 1 Department of Health Management, the Third Xiangya Hospital, Central South University, Tongzipo Road 138, Changsha, 410013 Hunan Province China; 2 Institute of Aviation Medicine, Beijing, China; 3 Statistics Department, Public Health Academy, Central South University, Changsha, Hunan China

**Keywords:** Arterial stiffness, Microalbuminuria, Metabolic syndrome

## Abstract

**Background:**

Increased arterial stiffness (iAS) and microalbuminuria (MAU), which may occur simultaneously or separately in the general population and share similar risk factors, are markers of macro- and microvascular injuries. Our research investigated the comorbidity of iAS and MAU in the middle-aged population and examined the heterogeneous effects of metabolic risk factors on iAS and MAU.

**Methods:**

We selected 11,911 individuals aged 45 to 60 years who underwent a health examination at the 3rd Xiangya Hospital between 2010 and 2014. Metabolic syndrome (MetS) was determined according to IDF/NHLBI/AHA-2009 criteria. Multinomial logistic regression was applied to evaluate the influence of MetS, components of MetS and clusters of MetS on the co-occurrence (MAU(+)/iAS(+)) or non-co-occurrence (MAU(+)/iAS(−) and MAU(−)/iAS(+)) of MAU and iAS.

**Results:**

Reference group was MAU(−)/iAS(−). A positive effect of MetS on the presence of MAU(+)/iAS(−), MAU(−)/iAS(+), or MAU(+)/iAS(+) is listed in ascending order based on odds ratios (ORs = 2.11, 2.41, 4.61, respectively; *P* < 0.05). Compared with MAU(+)/iAS(−), Elevated blood pressure (BP) (OR = 1.62 vs. 4.83, *P <* 0.05), triglycerides(TG) (OR = 1.20 vs. 1.37, *P <* 0.05) were more strongly associated with MAU(−)/iAS(+), whereas fasting blood glucose (FBG) was less associated (OR = 1.37 vs. 1.31, *P <* 0.05). Decreased high-density lipoprotein cholesterol(HDL-c) (OR = 1.84, *P <* 0.01) and elevated waist circumference(WC) (OR = 1.28 *P <* 0.01) were the most strongly associated with MAU(+)/iAS(−). Compared with the individuals without MetS, individuals with the elevated BP, FBG, TG and decreased HDL-c cluster had the greatest likelihood of presenting a MAU(−)/iAS(+) (OR = 5.98, *P* < 0.01) and MAU(+)/iAS(+) (OR = 13.17, *P* < 0.01), these likelihood was even greater than the cluster with simultaneous alteration in all five MetS components (OR = 3.89 and 10.77, respectively, *P* < 0.01), which showed the most strongly association with MAU(+)/iAS(+) (OR = 5.22, *P* < 0.01).

**Conclusion:**

Based on the heterogeneous influences of MetS-related risk factors on MAU and iAS, these influences could be selectively targeted to identify different types of vascular injuries.

## Background

Increased arterial stiffness (iAS) and microalbuminuria (MAU) are surrogate markers of macro [1] - and microvascular [2] damages, respectively; however, their comorbidity in the general population is unknown.

The formation of MAU is due to the leaking of small amounts of albumin from kidney into the urine [2], this reversible indicator is initiated by endothelial dysfunction and becomes fixed with the progression of renal microvascular abnormality [2]. iAS occurs as a consequence of biological aging [3] and arteriosclerosis [4], and pulse wave velocity is measured as a sign of macroangiopathy that reflects functional changes [5].

As two early markers of micro- and macrovascular complications [6, 7], there are connections between MAU and iAS: evidence has indicated that arterial stiffness is associated with MAU in patients with diabetes or hypertension [8] and in the general population [9, 10], and further study suggested that higher arterial stiffness is an independent risk factor for MAU [11]. Additionally, both iAS [12] and MAU [13] can be affected by similar factors: age [14–16]; gender [15, 17, 18]; and metabolic abnormalities, such as impaired glucose tolerance [16, 17, 19], high blood pressure [15, 16, 20], dyslipidemia [15, 16], and obesity [15, 21].

As manifestations of vascular damage in different areas [1, 2], since iAS and MAU have correlation and share common risk factors mentioned above, we hypothesize that taken together, iAS and MAU may represent a continuum in some populations; that is, individuals with less severe vascular disease express only one of these markers, while individuals with more severe disease express both. Systematic screening of this continuum is lacking in adults despite its potentially high utility in predicting vascular mortality; consequently, research regarding modifiable metabolic risk factors for MAU and iAS could be important for developing effective public health care initiatives.

The aims of our study were to measure the comorbidity of elevated MAU and iAS in the middle-aged population to determine whether MAU and iAS share a common pattern of individual risk factors for metabolic syndrome.

## Methods

### Study population and measurements

A cross-sectional study was conducted in a general population. The medical records of 11,911 participants from Central South China who underwent a health check at the Health Examination Management Center of the 3rd Xiangya Hospital from January 1, 2010, to October 31, 2014, were reviewed by trained staff. The inclusion criteria were as follows: age between 45 and 60 years and Han ethnicity. The exclusion criteria were heart failure, chronic nephritis, renal insufficiency, and pregnancy. The history of medication, smoking and cardiovascular disease was reviewed.

We only included middle-aged participants because vascular aging is common in older people [22]. This study was conducted according to the principles expressed in the Declaration of Helsinki and was approved by the Ethics Committee of the Third Xiangya Hospital, and the consent form was signed by each participant.

Waist circumference (WC) was measured by wrapping a measuring tape completely around the waist 0.5 cm above the belly button [23]. Systolic and diastolic blood pressure (SBP and DBP) was measured between 8 AM and 10 AM,following the guidelines from the American Health Association [24], all measurement were conducted using an automatic digital BP monitor(Omron 9020), with the participants seated after a 10-min rest period, with feet straight upon the ground and the back and arm supported, and with the antecubital fossa at the level of the heart. The maximum cuff inflation was calculated by adding 30 mmHg to the pulse obliteration pressure, and the cuff was deflated at a constant rate of 2 to 4 mmHg per second. Venous blood was collected in the morning after overnight fasting, serum samples were stored at 4 °C and were subject to testing (Hitachi 7170 s autoanalyzer) within 2 days, according to the instruction of analyzer, fasting blood glucose (FBG), triglycerides (TG), and high density lipoprotein cholesterol (HDL), cholesterol, uric acid, serum creatinine (Scr) were measured by enzymatic method using full-automatic biochemical analyser (Hitachi 7170 s). Estimated glomerular filtration rate [25] = 186 × Scr^− 1.154^ × age^− 0.203^ × 0.742 [if female].

All the participants’ morning urine specimens were tested. Urinary microalbumin was measured using the ELISA method. MAU < 20 mg/L was defined as normoalbuminuria, while ≥20 mg/L was defined as high MAU [26].

Brachial-ankle artery pulse wave velocity (baPWV) was used to evaluate arterial stiffness. After a 5-min rest in a supine position, baPWV was measured using the Colin-VP1000 (Omron, Japan) by trained nurses between 7 AM and 9 AM, with monitoring cuffs placed around both the upper and lower extremities, the data were recorded simultaneously by the instrument [27]. The time interval between the wave front of brachial waveform and that of ankle waveform was defined as the time interval between brachium and ankle (ΔTba). The path length from the heart to the brachium (Lb) was expressed using the following equation: Lb = 0.2195 × height of the patient (cm) − 2.0734. The path length from the heart to ankle (La) was expressed using the following equation: La = (0.8129 × height of the patient (cm) + 12.328). The baPWV was then calculated as follows: baPWV = (La/Lb)/Tba. baPWV≥1400 cm/s was considered high arterial stiffness [28].

### Definition of MetS and grouping

Definition of MetS is in accordance with the International Diabetes Federation/National Heart, Lung and Blood Institute/American Heart Association (IDF/NHLBI/AHA-2009) criteria, which are based on the presence of at least three of five risk factors (central obesity, elevated serum TG, low HDL, elevated BP, and elevated FBG). The cut-off values were as follows: 1) Central obesity, defined for Asian populations as WC ≥ 90 cm for males and WC ≥ 80 cm for females; 2) Elevated serum TG, defined as > 1.7 mmol/L; 3) Low HDL, defined as < 1.0 mmol/L for males and < 1.3 mmol/L for females; 4) Elevated BP, defined as systolic blood pressure (SBP) ≥130 or diastolic blood pressure(DBP) ≥85 mmHg or current use of anti-hypertensive medication; and 5) Elevated FBG, defined as ≥5.6 mmol/L or the current use of anti-diabetic medication.

Because MetS is defined by the presence of three or more altered components, participants with MetS have different combinations of the individual components of MetS (we abbreviated BP, FBG, WC, TG, HDL-c as B, G, W, T, H), such as WHT, BGWT, BGWTH.

All of the participants were divided into four groups according to the different combinations of MAU and baPWV values: 1) MAU(−)/iAS(−): baPWV< 1400 cm/s and MAU < 20 mg/L; 2) MAU(+)/iAS(−): MAU ≥ 20 mg/L and baPWV< 1400 cm/s; 3) MAU(−)/iAS(+): baPWV≥1400 cm/s and MAU < 20 mg/L; 4) MAU(+)/iAS(+): baPWV≥1400 cm/s and MAU ≥ 20 mg/L.

### Statistical analysis

SPSS 22.0 was applied. Continuous variables are expressed as mean ± standard deviation or interquartile range according to the distribution. Categorical variables are presented as percentages. Continuous variables between four groups were compared using one-way ANOVA or Kruskal-Wallis test, and categorical variables were compared using the Χ^2^ test. Multinomial logistic regression models (MLRM) for age, gender, uric acid, smoking status (never, former or current) and history of cardiovascular diseases (cerebrovascular events, coronary disease, peripheral arterial disease) and total cholesterol adjustment were used to explore the odds ratios of MAU(−)/iAS(+), MAU(+)/iAS(−),MAU(+)/iAS(+) based on MetS-related risk factors, including: 1) MetS itself, 2) The number of positive MetS components, 3) Individual MetS components, and 4) The clusters of MetS components. MAU(−)/iAS(−) was used as a reference. *P* < 0.05 was considered significant.

## Results

### Characteristics of the participants and prevalence of MetS components in the MAU(−)/iAS(−), MAU(+)/iAS(−), MAU(−)/iAS(+), MAU(+)/iAS(+) groups

**Fig. 1:**
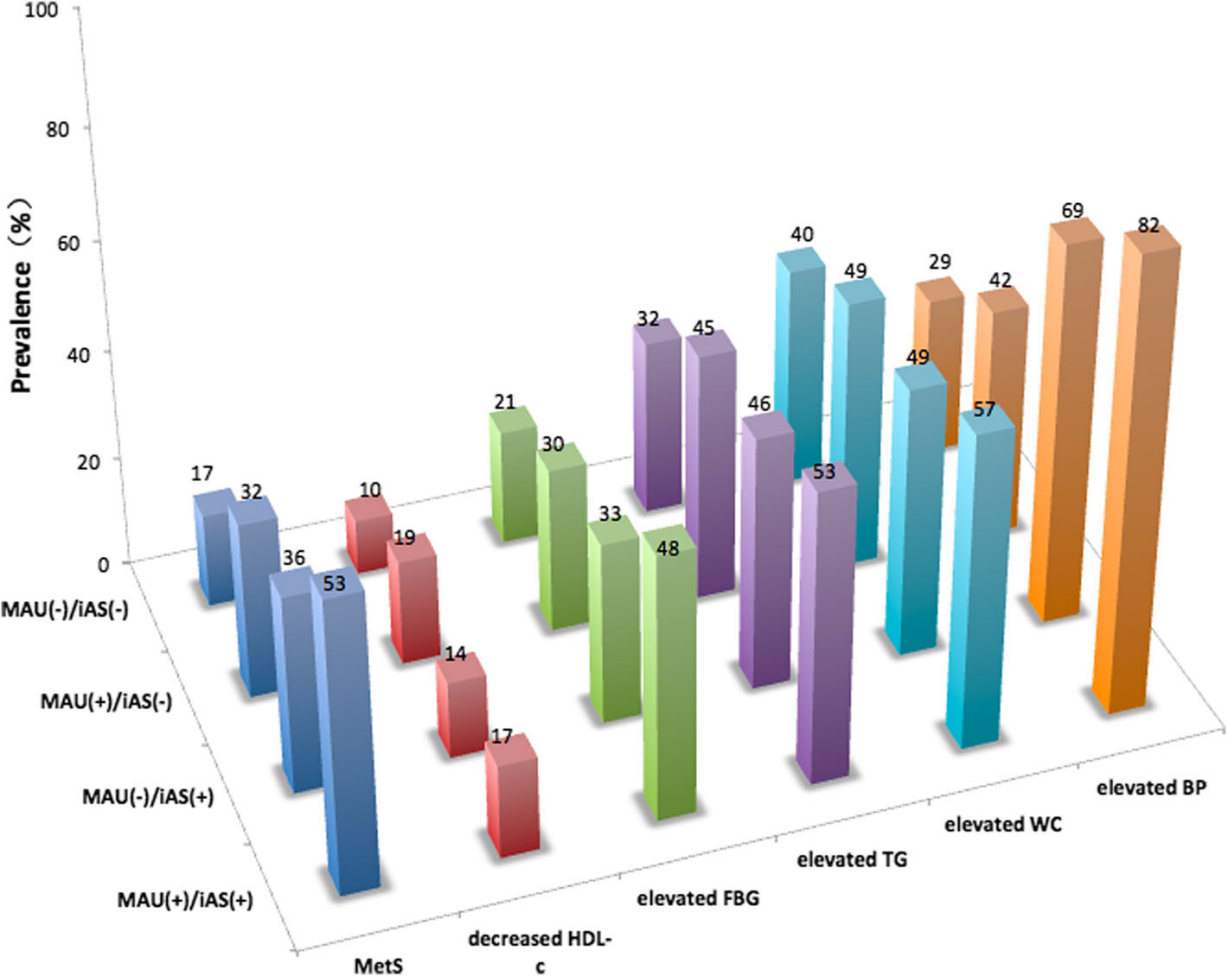
The prevalence of MetS components in the MAU(−)/iAS(−), MAU(+)/iAS(−), MAU(−)/iAS(+), and MAU(+)/iAS(+) groups

The clinical characteristics of 11,911 participants (52 ± 5.6 years) in this study are shown in Table 1.The prevalence of MAU(−)/iAS(−), MAU(+)/iAS(−), MAU(−)/iAS(+), MAU(+)/iAS(+) was 35%, 15%, 27%, and 23%. The proportions of elevated BP, FBG, WC, and TG were the highest, and the proportion of decreased HDL-c was the second highest in the MAU(+)/iAS(+) group; all of these parameters were proportionally the lowest in the MAU(−)/iAS(−) group. Compared with MAU(+)/iAS(−), the MAU(−)/iAS(+) group had higher proportion of elevated FBG, TG, BP and lower proportion of decreased HDL-c, whereas the proportions of elevated WC were equal in the MAU(+)/iAS(−) and MAU(−)/iAS(+) groups (Fig. 1).

**Table 1:** Characteristic of participants

Variables	Total *N* = 11,911	MA(−)/AS(−) *N* = 4228	MA(+)/AS(−) *N* = 1714	MA(−)/AS(+) *N* = 3238	MA(+)/AS(+) *N* = 2731	*P*
Age (years)	52 ± 5.6	50 ± 4.7	50 ± 4.6	54 ± 5.8	53 ± 5.9	0.000
Male	8105 (68.0%)	2610 (61.7%)	1320 (77.0%)	2078 (64.2%)	2097 (76.8%)	0.000
SBP (mmHg)	128 ± 17.2	118 ± 12.5	121 ± 13.1	134 ± 16.0	139 ± 17.4	0.000
DBP (mmHg)	81 ± 12.0	76 ± 9.8	79 ± 10.3	85 ± 11.1	89 ± 12.3	0.000
WC (cm)	86 ± 8.8	84 ± 9.9	86 ± 9.0	85 ± 8.3	88 ± 8.4	0.000
FBG (mmol/L)	5.2 (4.8,5.8)	5.1 (4.8,5.5)	5.1 (4.8,5.7)	5.3 (4.9,5.8)	5.5 (5.0,6.4)	0.000
TG (mmol/L)	1.5 (1.1.2.3)	1.4 (1.0,1.9)	1.6 (1.1,2.3)	1.6 (1.1,2.3)	1.8 (1.2,2.7)	0.000
HDL (mmol/L)	1.5 ± 0.4	1.5 ± 0.4	1.4 ± 0.4	1.5 ± 0.4	1.4 ± 0.4	0.000
Cholesterol (mmol/L)	5.3 ± 1.0	5.2 ± 0.9	5.1 ± 1.0	5.3 ± 1.0	5.3 ± 1.1	0.000
Uric acid (mmol/L)	320 ± 92	304 ± 90	325 ± 88	321 ± 92	340 ± 95	0.000
Non-smoker	6606 (55.5%)	2529 (59.8%)	833 (48.6%)	1901 (58.7%)	1343 (49.2%)	0.000
Antihyper- tensive	557 (4.7%)	162 (3.8%)	142 (8.3%)	135 (4.2%)	118 (4.3%)	0.000
Antidiabetic	365 (3.1%)	55 (1.3%)	45 (2.6%)	107 (3.3%)	158 (5.8%)	0.000
History of CVD	221 (1.9%)	42 (1.0%)	22 (1.3%)	74 (2.3%)	83 (3.0%)	0.000

Compared between four groups. Data was presented as proportion rate %, median (interquartile range) or mean ± standard deviation

### Components of MetS associated with MAU/iAS comorbidity

Table 2 refers to MAU(−)/iAS(−) as the reference group. Only elevated BP, FBG, and TG displayed the strongest associations with MAU(+)/iAS(+), the MAU(+)/iAS(−) group was associated with WC whereas MAU(−)/iAS(+) was not associated. There were higher risk of having elevated BP (OR = 4.83, *P <* 0.01 vs. 1.62, *P <* 0.01) and TG (OR = 1.37, *P <* 0.01 vs. 1.20, *P <* 0.05) in MAU(−)/iAS(+) compared with the risk in MAU(+)/iAS(−), conversely, lower risk of elevated FBG (OR = 1.31, *P <* 0.01 vs. 1.37, *P <* 0.01) in MAU(−)/iAS(+). Decreased HDL-c (OR = 1.84, *P <* 0.01 vs. 1.21, *P <* 0.05 and 1.49, *P <* 0.01) was the most strongly associated with MAU(+)/iAS(−) compared with MAU(−)/iAS(+) and MAU(+)/iAS(+) (Table 2).

**Table 2:** Association between MetS components and different comorbidities of iAS and MAU

Variables	MAU(−)/iAS(−)	MAU(+)/iAS(−)		MAU(−)/iAS(+)		MAU(+)/iAS(+)	
	OR	OR	95%CI	OR	95%CI	OR	95%CI
Elevated BP	1.0	1.62^**^	1.43–1.83	4.83^**^	4.35–5.37	8.67^**^	7.66–9.81
Elevated FBG	1.0	1.37^**^	1.20–1.57	1.31^**^	1.17–1.48	2.40^**^	2.13–2.71
Elevated WC	1.0	1.28^**^	1.13–1.44	0.98	0.88–1.09	1.24^**^	1.10–1.39
Elevated TG	1.0	1.20^*^	1.04–1.39	1.37^**^	1.21–1.56	1.41^**^	1.24–1.62
Decreased HDL-c	1.0	1.84^**^	1.54–2.19	1.21^*^	1.02–1.43	1.49^**^	1.26–1.77

*P < 0.05, **P < 0.01

### Number of MetS components associated with MAU/iAS comorbidity

A positive effect of MetS on the presence of MAU(+)/iAS(−), MAU(−)/iAS(+), or MAU(+)/iAS(+) is listed in ascending order based on odds ratios (OR = 2.11, 2.41, 4.61, respectively; *P* < 0.01). The ascending OR trend could also be observed when analyzing the influence of an increasing number of positive MetS components on MAU(+)/iAS(−), MAU(−)/iAS(+), and MAU(+)/iAS(+) (Table 3).

**Table 3:** Association between MetS or the number of positive MetS components and different comorbidities of MAU and iAS

Variables	MA(−)/AS(−)	MA(+)/AS(−)		MA(−)/AS(+)		MA(+)/AS(+)	
	OR	OR	95%CI	OR	95%CI	OR	95%CI
MetS	1.0	2.11^**^	1.84–2.41	2.41^**^	2.16–2.71	4.61^**^	4.11–5.18
Num. of MetS							
0 (ref)							
1	1.0	1.17	0.99–1.39	2.13^**^	1.81–2.50	3.25^**^	2.59–4.10
2	1.0	1.65^**^	1.38–1.96	3.98^**^	3.38–4.69	8.39^**^	6.69–10.52
3	1.0	2.33^**^	1.92–2.83	4.99^**^	4.87–5.98	15.85^**^	12.54–20.02
4	1.0	4.05^**^	3.14–5.22	7.35^**^	5.81–9.29	30.39^**^	23.10–39.98
5	1.0	6.16^**^	3.69–10.14	9.64^**^	6.06–15.33	46.65^**^	29.14–74.70

*P < 0.05, **P < 0.01

### Clusters of MetS components associated with MAU/iAS comorbidity

The frequency of the clusters (GHT, GWH, BWH, BGH, GWTH, BGWH) was low (less than 1%); therefore, we combined these clusters into an “other” category. Compared with the individuals without MetS, those with the BGTH cluster had the greatest likelihood of presenting a MAU(−)/iAS(+) (OR = 5.98, *P* < 0.01) or MAU(+)/iAS(+) (OR = 13.17, *P* < 0.01) profile; this likelihood was even greater than that associated simultaneous alteration in all five MetS components(BGWTH) (MAU(−)/iAS(+):OR = 3.89, *P* < 0.01; MAU(+)/iAS(+):OR = 10.77, *P* < 0.01). As the third most influential cluster for MAU(−)/iAS(+) and MAU(+)/iAS(+), the odds ratio for patients with BWG (MAU(−)/iAS(+): OR = 3.63, *P* < 0.01; MAU(+)/iAS(+): OR = 8,37, *P* < 0.01) was greater than that for patients with most of the clusters with four altered components. Regarding MAU(+)/iAS(−), the most influential clusters were BGWTH (OR = 5.22, *P* < 0.01), GWTH (OR = 4.08, *P* < 0.01) and BGW(OR = 3.40, *P* < 0.01), which are listed in descending order of ORs. WTH (OR = 2.45, *P* < 0.01), WGT (OR = 1.49, *P* < 0.05) and GWTH (OR = 4.08, *P* < 0.01) were associated with MAU(+)/iAS(−) but not with MAU(−)/iAS(+), whereas BTH was only associated with MAU(−)/iAS(+) (OR = 2.97, *P* < 0.01) and MAU(+)/iAS(+) (OR = 3.27, *P* < 0.01) (Table 4).

**Table 4:** Association between MetS clusters and different comorbidities of MAU and iAS

	Prevalence	MAU(−)/iAS(−)	MAU(+)/iAS(−)		MAU(−)/iAS(+)		MAU(+)/iAS(+)	
Variables	(n)	OR	OR	95%CI	OR	95%CI	OR	95%CI
WTH	158	1	2.45^**^	1.68–3.59	0.77	0.48–1.23	0.65	0.36–1.17
WGT	290	1	1.49^*^	1.06–2.10	0.98	0.71–1.34	1.25	0.88–1.76
BTH	129	1	1.42	0.72–2.78	2.97^**^	1.83–4.81	3.27^**^	1.95–5.48
BWT	800	1	1.67^**^	1.28–2.19	2.28^**^	1.84–2.82	3.79^**^	3.05–4.70
BGT	337	1	1.72^*^	1.05–2.82	3.13^**^	2.18–4.50	7.82^**^	5.52–11.08
BWG	507	1	2.65^**^	1.82–3.87	3.63^**^	2.67–4.93	8.37^*^	6.20–11.29
GWTH	112	1	4.08^**^	2.44–6.83	1.54	0.89–2.64	2.08^*^	1.16–3.74
BWTH	226	1	2.87^**^	1.81–4.57	2.98^**^	2.00–4.44	4.95^**^	3.32–7.43
BGTH	115	1	2.74^*^	1.11–6.80	5.98^**^	2.95–12.14	13.17^**^	6.59–26.32
BGWT	714	1	3.40^**^	2.49–4.64	2.94^**^	2.24–3.86	8.20^**^	6.31–10.64
BGWTH	263	1	5.22^**^	3.18–8.57	3.89^**^	2.48–6.11	10.77^**^	6.97–16.63
Others	270	1	2.40^**^	1.59–3.63	2.28^**^	1.57–3.31	5.02^**^	3.53–7.15

W: increased waist circumference, T: increased triglycerides, B: increased blood pressure, G: increased fasting blood glucose, H: decreased HDL-c. *P < 0.05, **P < 0.01

## Discussion

This current study has shown the heterogeneous association between cross-combination of MAU/iAS and MetS itself, the number of positive MetS components, individual MetS components, the clusters of MetS components.

There are close relation between iAS or MAU to the prediction and development of cardiovascular diseases [29, 30]. The influence of MetS on arterial stiffness or MAU has clearly been demonstrated [31–33]. The novelty of our study is that we combined the study of iAS and MAU, which both reflect vascular organ damage, and found that MetS-related risk factors heterogeneously contributed to phenotypes of vascular damage to different body parts. To reduce the influence of age, which is an essential factor in vascular aging, we chose to examine a middle-aged population [34]. We used MLRM instead of the standard logistic regression model (SLRM), which requires that MAU and arterial stiffness be considered separately as dependent variables; however, a limitation of the SLRM its the lower efficacy. For instance, when the predictors of MAU were analyzed, the reference category (i.e., no increase in MAU) consisted of individuals who may suffer from iAS; this may have resulted in a confounding bias in the estimation of risk factors’ effects on MAU because high MAU and iAS might share similar risk factors. The same principle applied to the analysis of risk factors of arterial stiffness. Furthermore, the MLRM is more sophisticated, allowing the simultaneous estimation of the risk factors’ effects on both the presence of and the association between MAU and iAS without bias arising from the non-independence of the outcomes [35].

Consistant with our hypothesis that iAS and MAU may represent a continuum in some populations with more severe vascular while individuals with less severe vascular disease express only MAU or iAS, high microalbuminuria and arterial stiffness coexistd in a substantial percentage of individuals who exposed to relatively higher degree of MetS, which suggested increased propensity towards worse vascular condition [36].

Previous studies have reported that an increasing number of MetS components has an increasingly negative effect on iAS [37] or MAU [38]. According to MLRM, the overall risk of having MAU alone was lower than that of having iAS alone with regard to the effect of metabolic disturbances, regardless of the number of MetS components or the presence of MetS. When analyzing individual components, we found that elevated BP was the main distinguishing factor, contributing to a much higher risk of arterial stiffness alone. The ORs for the risk of elevated BP associated with baPWV in a Chinese population were between 5.4 (OR for males) and 12.6 (OR for females) [39]. The risks of having high baPWV with or without MAU in our results were within or below this range and more accurately revealed the different associations between blood pressure and arterial stiffness in the presence of confounding factors. Our research revealed that abdominal obesity was not associated with baPWV alone. Studies have reported a negative association between obesity and vascular stiffness in young people [40, 41], but a positive association was reported in older adults and the elderly [42]. The physiological mechanisms linking body fat with arterial stiffness are not fully understood; however, we speculated that with increases in age, body fat becomes increasingly more positively associated with PWV [43], and middle age is a turning point. Mechanism of MetS-related arterial stiffness and microalbuminuria elevation involves oxidative, nitrosative stress [44, 45], and inflammation [46], for example, via these products, hyperglycemia increases endothelial [47] and glomerular filtration barrier permeability [48], and accelerates arterial stiffness [49], inversely, antioxidant effects of HDL-c contributes to the vascular protection [50].

Previous studies examined the clusters of altered MetS components associated with the burden of large arteries [12, 51], while the current study reported the simultaneous impact of specific MetS clusters on the heterogeneity of phenotypes of macro- and microvascular abnormalities. The clusters identified as the three most significant determinants of stiff arteries alone and comorbid stiff arteries and microalbuminuria were similar but differed from the determinants of microalbuminuria alone. Moreover, containing B and G was necessary for the clusters to positively impact iAS and MAU, respectively. In light of this disparity, it was possible for us to predict macro- or microvascular complications according to the different predictive powers of specific MetS clusters and to provide specific follow up for patients who are susceptible to these predicted vascular diseases.

The first limitation of our study was that we report analysis from cross-sectional data only. Therefore, we cannot conclude about the role of MetS-related risk factors as potential predictiors of future events (nor was this a goal of the present study). Secondly, baPWV has been criticized to span a more heterogeneous arterial tree than carotid-femoral pulse wave velocity (cfPWV), thus subsumes greater heterogeneity, cfPWV has been more accurate and reliable [52, 53], however, as the target arteries may be somewhat difficult to be perceived by probes, the utilization of this cfPWV is lower than baPWV [53]. Since there was a strong positive association between cfPWV and baPWV [54], we assume that the result is also reliable.

This observational study suggested that MetS itself, the number of positive MetS components, individual MetS components, and the clusters of MetS components showed different patterns of associations with MAU or iAS. According to this study, we present the specifically protective pathway of vascular complication, for example, microvascular impairment rather than macrovascular stiffness should be more closely monitored in people with combination of elevated WC, TG, blood pressure. In summary, these heterogeneous influences of MetS-related risk factors suggest possible preventive intervention: identifying, assessing, and following up more selectively individuals with specific metabolic disturbance may facilitate effective prevention of types of vascular injuries.

## Conclusion

Heterogeneous association exist between MetS and cross-combination of MAU/iAS, based on these heterogeneous influences of MetS-related risk factors on MAU and iAS, identifying different types of vascular injuries could be selectively targeted.

## Abbreviations

baPWV: Brachial-ankle artery pulse wave velocity
BP: Blood pressure
FBG: fasting blood glucose
HDL: high density lipoprotein cholesterol
iAS: Increased arterial stiffness
MAU: microalbuminuria
MetS: metabolic syndrome
MLRM: Multinomial logistic regression models
SLRM: standard logistic regression model
TG: triglycerides
WC: waist circumference
